# A Survey of Oral Assessment and Healthcare Education at Nursing Schools in Japan

**DOI:** 10.1016/j.identj.2022.09.006

**Published:** 2022-11-05

**Authors:** Satoru Haresaku, Keiko Kubota, Maki Miyoshi, Mika Obuse, Hisae Aoki, Fuyuko Nakashima, Masumi Muramatsu, Hitomi Maeda, Souhei Uchida, Mami Miyazono, Hidechika Iino, Toru Naito

**Affiliations:** a Department of Nursing, Fukuoka Nursing College, Fukuoka, Japan; b Graduate School of Nursing Science, St. Luke's International University, Chuo-ku, Tokyo, Japan; c School of Nursing, Sapporo City University, Sapporo, Hokkaido, Japan; d Department of Nursing, Faculty of Life Sciences, Kumamoto University, Chuo-ku, Kumamoto, Japan; e Section of Geriatric Dentistry, Department of General Dentistry, Fukuoka Dental College, Sawara-ku, Fukuoka, Japan

**Keywords:** Nursing student, Oral assessment, Multiprofessional education, Oral health care education, Nationwide survey, Bachelor of nursing curriculum

## Abstract

**Objectives:**

This study aimed to investigate the performance of oral assessment and health care education at nursing schools across Japan to identify problems and the need for oral health professional support.

**Methods:**

The participants were the academic staff in charge of oral health care education in the basic, adult, gerontological, and home nursing fields of 295 schools in Japan that offered a bachelor's degree in nursing. A questionnaire was sent to 1180 heads of the nursing fields of these schools. They were requested to have oral health care educators complete the survey; data on the performance of oral assessment and health care education and human resources allocated were collected through a questionnaire.

**Results:**

A total of 311 (26%) questionnaires were returned, 196 (63%) of which were completed by oral health care educators. Regarding the performance of oral assessment education, the majority (71%) of them spent less than 2 hours in teaching, and only 24.5% taught the usage of oral assessment tools. Regarding the performance of oral health care education, more than 90% spent less than 2 hours in lecture-based and practical oral health care education, respectively. Less than half taught the association of periodontal diseases with diabetes and cardiovascular diseases and use of fluoride for caries prevention in the lectures, and only approximately 30% taught the usage of an interspace brush or dental floss. Moreover, less than 10% of oral health professionals taught oral health care in lectures or practical oral health care.

**Conclusions:**

This study revealed problems associated with oral assessment and health care education in Japanese nursing schools. To address these, support from oral health care professionals is required. Further studies are also required to reveal problems in oral assessment and health care education in other nursing schools that do not offer a bachelor's degree programme in Japan and other countries.

## Introduction

The population in Japan is ageing at a rapid pace. The elderly population (>65 years) increased from 21.7 million in 2000 to 33.1 million in 2015.^1^ Moreover, the number of elderly people who used long-term care services increased from 0.52 million to 0.90 million in facilities and 0.97 million to 3.82 million at home during the same period. The number of patients with dementia is expected to increase from 4.6 million in 2012 to 7.0 million in 2025.^1^

Oral health care and treatment are effective in preventing not only dental but also systemic diseases such as aspiration and ventilator-associated pneumonia, cardiovascular disease, and diabetes in elderly people.^2^, ^3^, ^4^ Additionally, elderly patients in hospitals and community-dwelling frail elderly people who have difficulties of visiting dental clinics are more likely to have dental diseases including eating and swallowing disorders than healthy adults.^5^, ^6^, ^7^, ^8^

Nurses play an important role in caring for elderly people and performing oral assessments, dental referrals through physicians, and collaborative oral health care practices with oral health professionals.^9^, ^10^, ^11^

However, some nurses and nursing students may have poor knowledge and negative attitudes towards oral assessment and health care.^12^, ^13^, ^14^ Regarding this, multiprofessional education was effective in improving their knowledge and attitudes on oral assessment^15^ or health care.^16^, ^17^, ^18^, ^19^, ^20^ However, only the studies’ participants benefitted from the education. Therefore, nursing curricula across nursing schools must be enhanced to improve the performance of oral assessment and health care of nurses as a whole. Several studies have suggested that oral assessment and health care education curricula in nursing schools be made more extensive.^16^^,^^17^^,^^19^, ^20^, ^21^, ^22^, ^23^ However, no study in Japan has investigated these curricula on a nationwide scale.

Therefore, this study aimed to investigate the status of oral assessment and health care education at nursing schools across Japan to identify the problems and need for oral health professional support.

## Material and methods

### Design and sample

A nationwide cross-sectional study was conducted using a self-administered questionnaire for the academic staff in charge of oral health care education in the basic, adult, gerontological, and home nursing fields of 4-year nursing schools with a bachelor of nursing programme in Japan between May 11 and July 10, 2022. Those fields were selected because they were considered fields in which teaching nursing students how to perform oral assessment and health care amongst middle-aged and elderly patients is important.

Four copies of the questionnaire with 4 return envelopes were sent to all the heads of the 4-year nursing schools by mail. They were asked to send the questionnaires to each head of the 4 nursing fields. These heads were then asked whether an appointed academic staff member in their fields was in charge of oral health care assessment and education (hereafter referred to as an “oral health care educator”; Appendix 1). If an educator was employed, the heads were requested to have the educator complete the questionnaire and return it in the attached return envelopes. If no educator was employed, they were requested to choose “There is no academic staff in charge of oral health care education in my field” and return the questionnaire (without completing it) in the enclosed return envelopes.

### Ethical considerations

The purpose of the study was explained to the head of the schools and nursing fields and participants through the documents enclosed with the questionnaire. A returned questionnaire was considered consent to participate. This study was approved by the ethics committee of Fukuoka Gakuen, Japan (approval #588).

### Instrument

The questionnaire was derived from a previous one that was used for evaluating the effect of oral health care education by multi-interprofessional on nursing students’ knowledge, attitudes, and confidence in performing oral health care.^24^ Prior to the study, the items in the questionnaire were discussed with 5 nursing academic staff who worked in nursing schools and were engaged in oral assessment and health care education for more than 10 years. Furthermore, the content of the questionnaire was checked by 2 dental academic staff who were employed in nursing and dental schools. Cronbach alpha values for each domain ranged from 0.639 to 0.760.

The questionnaire items were divided into the following sections: length of work experience as oral health care educators, oral assessment education, oral health care education in lectures, practical oral health care education, and human resources of oral health care education (Appendix 1).

Q2, Q5, and Q7 assessed how many minutes the participants spent imparting oral assessment education, lectures on oral health care, and practical oral health care instructions in their nursing fields, respectively. The times reported were converted from minutes to hours. Responses to Q2 were categorised as follows: 0 (none), <1 hour (except 0), 1–2 hours, 2–3 hours, 3–4 hours, and >4 hours. Furthermore, responses to Q5 and Q7 were categorised as follows: 0 (none), <1 hour (except 0), 1–2 hours, 2–3 hours, and 3–4 hours.

### Data collection

The questionnaire was distributed to 1180 participants in 295 nursing schools. Out of the total, 311 questionnaires were returned, and the response rate was 26.4%.

### Data analysis

The chi-square test was used to compare the differences in the performances of oral assessment and health care education and human resources of oral health care amongst the nursing fields. Data were analysed at a 5% significance level. Statistical analyses were performed using IBM SPSS Statistics for Windows, version 28 (IBM Corporation).

## Results

A total of 311 (26.4%) questionnaires were retuned. Amongst the respondents, 196 (63.0%) completed the questionnaire as oral health care educators, whilst 115 (37.0%) responded with, “There are no oral health care educators in my field” and did not complete it (Table 1). Consequently, the percentages of educators who completed the questionnaire in the basic, adult, gerontological, and home nursing fields amongst the respondents were 94.6%, 34.2%, 87.0%, and 30.0%, respectively.

**Table 1:** Number (%) of participants responding whether there were oral health care educators in their nursing field and number (%) of oral health care educators who completed the questionnaire.

Field	Total	Basic nursing	Adult nursing	Gerontological nursing	Home nursing
Number of fields	1180	295	295	295	295
Respondent (%)	311 (26.4)	93 (31.5)	79 (26.8)	69 (23.4)	70 (23.7)
Oral health care educator (%)*	196 (63.0)	88 (94.6)	27 (34.2)	60 (87.0)	21 (30.0)

⁎ The percentages are the rate of oral health care educator in responders.

Regarding the length of their work experience as oral health care educators, 135 (70.3%) answered “less than 10 years,” 43 (22.4%) answered “10–20 years,” and 14 (7.3%) answered “more than 20 years.”

Table 2 shows the distribution of hours and usage of tools in oral assessment education according to nursing fields. Regarding the question on how many hours they spend on oral assessment education in their nursing field, 5.1% answered “none,” 35.2% answered “<1 hour (except none),” 31.1% answered “1–2 hours,” and the rest (28.5%) answered “>2 hours.”

**Table 2:** Distribution (%) of hours and usage of oral assessment tools in oral assessment education according to nursing fields.

Nursing field	Total (n = 195)	Basic nursing (n = 87)	Adult nursing (n = 27)	Gerontological nursing (n = 60)	Home nursing (n = 21)	*P* value*
How many minutes† do you spend imparting education on oral assessment in your nursing field?						
0 (none)	5.1	3.4	14.8	3.3	4.8	.230
<1 h (except 0)	35.2	33.0	51.9	28.3	42.9	
1–2 h	31.1	30.7	18.5	35.0	38.1	
2–3 h	5.6	6.8	0.0	6.7	4.8	
3–4 h	15.8	15.9	14.8	18.3	9.5	
>4 h	7.1	10.2	0.0	8.3	0.0	
Do you provide instructions on the usage of oral assessment tools in oral assessment education?						
Yes	24.5	20.5	29.6	31.7	14.3	.260
No	75.5	79.5	70.4	68.3	85.7	
What types of oral assessment tools are covered in the education? (multiple choice)						
Oral Health Assessment Tool (OHAT)	14.3	17.0	11.1	11.7	14.3	.776
Oral Assessment Guide (OAG) including Revised Oral Assessment Guide (ROAG)	12.8	5.7	18.5	23.3	4.8	.007
Other oral assessment tools	1.5	1.1	7.4	0.0	0.0	.057

⁎ Chi-squared test.

† The times reported were converted from minutes to hours.

Amongst the respondents, only 24.5% taught the usage of oral assessment tools, 14.3% taught the usage of the Oral Assessment Tool (OHAT), and 12.8% taught the usage of the Oral Assessment Guide (OAG) including the Revised Oral Assessment Guide (ROAG). A significant difference was observed in the usage of the OAG amongst the nursing fields.

Table 3 shows the distribution of hours and the content of lectures regarding oral health care education classified according to the nursing fields. Regarding how many hours participants spend on lectures regarding oral health care in their nursing field, 5.1% answered “none,” the majority (56.6%) answered “< 1 hour (except none),” and the rest (38.3%) answered “>1 hour.” Approximately 20% in the adult nursing field and 10% in the home nursing field stated that they had no time to teach oral health care. A significant difference was observed in the distribution of hours amongst the nursing fields (*P* < .05).

**Table 3:** Distribution (%) of hours and content of lectures regarding oral health care education according to nursing fields.

Nursing field	Total (n = 195)	Basic nursing (n = 87)	Adult nursing (n = 27)	Gerontological nursing (n = 60)	Home nursing (n = 21)	*P* value*
How many minutes† do you spend imparting lectures on oral health care in your nursing field?						
0 (none)	5.1	2.3	11.1	1.7	19.0	.027
<1 h (except 0)	56.6	61.4	59.3	51.7	47.6	
1–2 h	33.2	33.0	29.6	36.7	28.6	
2–3 h	5.1	3.4	0.0	10.0	4.8	
>3 h	0.0	0.0	0.0	0.0	0.0	
What is taught in the lectures on oral health care in your nursing field? (multiple choice)						
Preventive effect of oral health care on aspiration pneumonia	99.0	100.0	100.0	98.3	94.7	.192
Preventive effect of oral health care on periodontal disease	68.8	75.6	42.3	67.8	77.8	.011
Preventive effect of oral health care on dental caries	64.4	75.9	30.8	63.2	61.1	<.000
Oral hypofunction	57.1	47.7	42.3	71.7	73.7	.005
Oral frailty	54.7	38.8	34.6	83.3	63.2	<.000
Association between periodontal disease and diabetes	48.1	39.3	69.2	46.6	63.2	.029
Association between periodontal disease and cardiovascular disease	43.8	28.0	73.1	46.6	63.2	<.000
Usage of fluoride for dental caries prevention	21.5	21.4	11.5	25.4	23.5	.550
Perioperative oral function management	18.3	10.8	65.4	11.9	5.6	<.000
Oral diadochokinesis	12.3	6.0	11.5	18.6	22.2	.072

⁎ Chi-squared test.

† The times reported were converted from minutes to hours.

Almost all of them taught the preventive effect of oral health care on aspiration pneumonia; however, less than 70% taught the preventive effect on periodontal diseases and dental caries. More than half taught oral hypofunction and frailty, although less than 50% taught the association of periodontal disease with diabetes and cardiovascular disease. Moreover, less than a quarter of them taught the use of fluoride, perioperative oral function management, and oral diadochokinesis. Except for the preventive effect on aspiration pneumonia, use of fluoride, and oral diadochokinesis, significant differences were observed in what they taught in lectures regarding oral health care amongst the nursing fields (*P* < .05–.001).

Table 4 shows the distribution of hours and the content of practical oral health care education classified according to nursing fields. Regarding how many hours they spend on practical oral health care in their nursing field, 24.5% answered “none,” 39.3% responded “<1 hour (except none),” and others (36.1%) answered “more than 1 hour.” More than half in the adult and home nursing fields stated that they had no time to teach about practical oral health care. A significant difference was observed in the distribution of hours amongst the nursing fields (*P* < .001).

**Table 4:** Distribution (%) of hours and content of practical oral health care education according to nursing fields.

Nursing field	Total (n = 195)	Basic nursing (n = 87)	Adult nursing (n = 27)	Gerontological nursing (n = 60)	Home nursing (n = 21)	*P* value*
How many minutes† do you spend on imparting practical oral health care education in your nursing field to your students?						
0 (none)	24.5	13.6	59.3	15.0	52.4	<.000
<1 h (except 0)	39.3	45.5	22.2	40.0	33.3	
1–2 h	29.0	30.0	19.0	40.0	10.0	
2–3 h	1.5	3.4	0.0	0.0	0.0	
>3 h	5.6	8.0	0.0	5.0	4.8	
What practical techniques in oral health care education are addressed? (multiple choice)						
Usage of a dental sponge	81.3	92.9	26.1	88.1	76.2	<.000
Toothbrushing technique	73.7	94.0	21.7	70.7	57.1	<.000
Denture removal, cleaning, and replacement	72.4	69.5	26.1	94.9	71.4	<.000
Usage of personal protective equipment (PPE) during oral care practice for patients	69.9	91.6	52.2	57.6	38.1	<.000
Oral function training (oral exercise)	59.9	38.1	43.5	89.8	81.0	<.000
Salivary gland massage	56.8	39.8	36.4	83.1	71.4	<.000
Usage of an interspace brush	29.3	34.9	4.3	29.3	35.0	.037
Infection prevention measures during oral health care practice for patients other than PPE	27.6	28.0	21.7	27.1	33.3	.860
Usage of a dental floss	27.5	32.1	4.3	27.6	35.0	.055
Usage of a portable suction machine for oral health care	27.4	19.3	30.4	27.1	57.1	.007

⁎ Chi-squared test.

† The times reported were converted from minutes to hours.

More than 70% of the educators taught basic skills such as the usage of a dental sponge, toothbrushing technique, and denture removal, cleaning, and replacement; however, less than 30% taught the usage of an interspace brush and dental floss. More than half taught the training of oral function (oral exercise) and salivary gland massage, and approximately 70% taught the usage of personal protective equipment (PPE).

More than 90% taught the usage of a dental sponge and PPE and toothbrushing technique in the basic nursing fields, and more than 90% taught denture removal, cleaning, and replacement in the gerontological nursing fields. The percentages in those fields were more likely to be higher than in other fields. Except for the infection prevention measures and usage of dental floss, significant differences were observed in what they taught in practical oral health care education amongst the nursing fields (*P* < .05–.001).

Table 5 shows the human resources for oral health care education classified according to the nursing fields. Amongst them, only approximately 5% of dentists and dental hygienists taught oral health care in lectures, and 1.0% of dentists and 6.1% of dental hygienists taught practical oral health care in the nursing fields. Significant differences were not observed in the human resources of oral health professionals for education amongst the fields.

**Table 5:** Human resources of oral health care education according to nursing fields.

Nursing field	Total (n = 195)	Basic nursing (n = 87)	Adult nursing (n = 27)	Gerontological nursing (n = 60)	Home nursing (n = 21)	
	N (%)					*P* value*
Who teaches oral health care in lectures in your nursing fields? (multiple choice)						
Nurse	189 (96.4)	87 (98.9)	24 (88.9)	59 (98.3)	19 (90.5)	.033
Dentist	10 (5.1)	1 (1.1)	2 (7.4)	6 (10.0)	1 (4.8)	.105
Dental hygienist	11 (5.6)	3 (3.4)	2 (7.4)	5 (8.3)	1 (4.8)	.607
Speech-language-hearing therapist	1 (0.5)	0 (0.0)	0 (0.0)	1 (1.7)	0 (0.0)	.517
Others	3 (1.5)	2 (2.3)	0 (0.0)	1 (1.7)	0 (0.0)	.783
Who teaches practical oral health care in your nursing fields? (multiple choice)						
Nurse	158 (80.6)	80 (90.9)	12 (44.4)	54 (90.0)	12 (57.1)	<.000
Dentist	2 (1.0)	0 (0.0)	0 (0.0)	2 (3.3)	0 (0.0)	.205
Dental hygienist	12 (6.1)	3 (3.4)	1 (3.7)	7 (11.7)	1 (4.8)	.197
Speech-language-hearing therapist	2 (1.0)	0 (0.0)	1 (3.7)	0 (0.0)	1 (4.8)	.095
Others	9 (4.6)	2 (2.3)	0 (0.0)	5 (8.3)	2 (9.5)	.141

⁎ Chi-squared test.

## Discussion

This study is the first attempt to explore the performance of oral assessment and health care education in the basic, adult, gerontological, and home nursing fields of nursing schools by conducting a nationwide questionnaire survey.

This study revealed the low performance time of oral assessment education and low usage of oral assessment tools. The OHAT and OAG were developed by dentists to promote other health professionals’ performance of oral assessment for patients.^24^^,^^25^ The last evaluation item in OHAT is “whether the patient needed a dental referral.”^24^ A study reported that the nurses’ oral assessment performance and knowledge of the usage of oral assessment tools were significantly associated with their dental referral performance.^11^ Therefore, promoting the nurses’ performance of oral assessment and usage of tools may contribute to more dental referral of patients. However, a study showed that approximately 20% to 40% of nurses performed oral assessments for more than 50% of their inpatients in hospitals, and the percentages of OHAT and OAG usage in hospitals were 24.7% and 10.0%, respectively.^14^ Moreover, another study showed that only approximately half of nurses performed oral health checkups for elderly patients.^26^ Therefore, the low performance of oral assessment education for nursing students might negatively affect their performance of oral assessment in patients after their qualification. A study regarding oral assessment education for nursing students reported that multiprofessional oral assessment education for nursing students was effective in improving their attitudes, confidence, abilities, and performance of oral assessment.^15^ Therefore, oral health professionals should educate nurses regarding the importance of performing oral assessment in patients with tools. Moreover, dentists must also participate, if possible, in this education to promote dental referrals and treatment and collaborative oral health care.

This study revealed that the performance of oral health care in the nursing fields was also low. More than 90% spent less than 2 hours on oral health care education in lectures and practical oral health care education, respectively. In addition, this study showed that the percentages of the educators who teach about the preventive effects of oral health care on dental diseases, the association of periodontal with systemic diseases, and the use of fluoride for caries prevention were lower than the percentage of those who teach the preventive effect on aspiration pneumonia. Periodontal disease and dental caries are the main causes of tooth loss^27^ and contribute to oral hypofunction^28^ and frailty,^29^ dementia,^30^ and pneumonia mortality.^31^ Moreover, periodontal disease is associated with systemic diseases such as cardiovascular disease^32^ and diabetes.^33^ Some studies showed that the provision of oral treatment by oral health professionals such as the removal of dental calculus and usage of fluoride gel are effective in the prevention of dental diseases in elderly patients.^34^^,^^35^ Therefore, oral health professionals must educate both oral health educators and nursing students about the importance of the prevention of dental diseases that negatively affect the patients’ oral function, systemic disease, and quality of life.

This study showed that less than 30% of oral health educators taught the usage of an interspace brush and dental floss, although the percentages of them teaching about the usage of other basic oral health care tools and PPE were high. Interdental cleaning tools are effective in removing dental plaque and food debris in interdental spaces. A few studies showed that toothbrushing and using an interspace brush were more effective in decreasing plaque and gingivitis than toothbrushing alone.^36^ However, a previous study in a Japanese hospital showed that only 2% of nurses used interdental cleaning tools in their oral health care practice with patients.^37^ Therefore, oral health professionals should encourage educators to teach about the usage of interdental cleaning tools in practical oral health care education in order to increase the likelihood of their usage amongst patients by nursing students after qualification.

Oral frailty was defined by the Japan Dental Association in May 2019, and oral hypofunction was defined by the Japanese Society of Gerodontology.^38^ Moreover, oral frailty and hypofunction are related.^38^ This study revealed for the first time that more than half of educators taught oral hypofunction and frailty, oral function training, and salivary gland massage. Therefore, oral frailty and hypofunction are increasingly being taught in nursing education.

Collaborative oral health care by oral health professionals and nurses for patients in hospitals or frail elderly persons in long-term care facilities and at home is important to prevent pneumonia and cardiovascular disease.^3^^,^^32^ Therefore, its importance should be taught in nursing education. However, this study showed that educators stated that they do not have enough time to teach about oral health care; moreover, the human resources of oral health professionals were low. Some studies showed that multiprofessional education for nursing students was effective in improving their knowledge and attitudes on oral health care practice for patients.^15^, ^16^, ^17^, ^18^, ^19^, ^20^ Moreover, one of the studies showed not only that multiprofessional education was effective in improving the nursing students’ perception of the importance of collaborative oral health care practice but also that the level of perception amongst nursing students became higher than dental students after the education.^20^ Therefore, oral health professionals should participate in the education of nursing students in order to help them perceive the importance of oral health care and perform collaborative oral health care with patients after their qualification.

Statistical analysis revealed significant differences in the performance of oral assessment and health care education amongst different nursing fields. Thus, oral health care professionals should impart education on oral assessment and health care to both educators and nursing students.

Nevertheless, this study has several limitations. First, the response rate was only 26.4%, although all Japanese nursing schools with a bachelor of nursing programme were selected as participants. A study regarding the response rates of mail surveys showed that response rates were higher when the survey's topic was interesting to the respondents.^39^ Therefore, the respondents in this study may be more interested in oral assessment and health care education than the nonrespondents. In addition, in order to investigate the content of oral assessment and health care education in detail, only the educator responsible for oral health care education in the nursing fields may complete the questionnaire. Therefore, the performance of oral assessment and health care education amongst the nursing fields across Japan might be lower than the nursing fields in this study. Only 4 nursing fields were investigated, and other fields such as basic medical science, maternal nursing, paediatric nursing, and psychiatric nursing were not included. Oral assessment and health care may be taught in those fields, too. However, the education of nursing students in the fields investigated in this study was thought to be more important for the promotion of collaborative oral health care for middle-aged and elderly patients. Almost all Japanese schools that offer a bachelor's degree programme in nursing impart education in basic, adult, gerontological, and home nursing; however, such fields may not exist in other countries. Therefore, when problems of education according to nursing fields in other countries are investigated and compared amongst nursing fields, the choice of nursing field may need to be considered. Less than 35% of the educators in the adult and home nursing fields participated in this study; the low percentage of participants in these fields might have influenced the differences in the performance of education and in human resources amongst the nursing fields in the study. In Japan, there are 1088 nursing schools (including those that do not offer a bachelor's degree programme in nursing).^40^ The performance of oral assessment and health care education might be lower in nursing schools without a bachelor's degree programme than in those with a bachelor's degree programme due to differences in the duration of their curricula (3 vs 4 years). However, further studies are needed to confirm this hypothesis. Oral health care programmes in nursing schools have been investigated in other countries^16^^,^^17^^,^^19^^,^^21^, ^22^, ^23^; findings from these investigations have suggested the need for oral health care education for nursing students. However, only few schools have been investigated; a worldwide survey is needed to investigate problems in ageing societies on a more global scale. Moreover, oral assessment and health care education are important not only for nursing students and nurses but also for other health care professionals and students. Therefore, further studies are needed to investigate the problems associated with oral assessment and health care education for health care professionals and students worldwide.

## Conclusions

Our findings revealed several problems with oral assessment and health care education in Japanese schools that offer a bachelor's degree programme in nursing. These include low percentages of educators teaching nursing students about the use of oral assessment tools, the importance of preventing dental diseases, the use of interspace cleaning tools, and the importance of having oral health care professionals as educators of oral health care. Our findings have also shown the need for support from oral health care professionals.

## Author Contributions

S.H. searched and reviewed the literature, analysed the data, and wrote the manuscript. M.M., H.A., F.N., and S.U. assisted in finding documents, issuing questionnaires, analysing data, and examining the manuscript. M.O., M.M., and H.M. assisted in making the questionnaire in this study and advised on conducting the investigations in 295 nursing schools. K.K., M.M., H.I., and N.T. critically reviewed the manuscript and supervised the whole study process. All authors read and approved the final manuscript.

## Conflict of interest

None disclosed.

## Appendix 1

### Appendix 1 The content of questionnaire survey

To a head of your nursing fields

Are there any academic staff in charge of oral health care education is employed in your field?

Yes. → Please ask the staff to complete this questionnaire and return it in the enclosed return envelope.

No, there is no academic staff in charge of oral health care education in my field. → Please return this questionnaire without completing it in the enclosed return envelope.

To a staff in charge of oral health care education

Please complete the questions in Q1–10

Q1: How many years you have worked as an oral care educator? Please choose one and check it.

Answer: 0–4 years/5–9 years/10–14 years/15–19 years/≧20 years

Q2: How many minutes do you spend on oral assessment education in your nursing field?

Answer: () minutes

Q3: Do you instruct on the usage of oral assessment tools in oral assessment education?

Answer: Yes/No

Q4: What kinds of oral assessment tools are used in the education? (multiple choice)

Oral Health Assessment Tool (OHAT)/Oral Assessment Guide (OAG) including Revised Oral Assessment Guide (ROAG)/Other oral assessment tools

Q5: How many minutes do you spend the lectures regarding oral health care in your nursing field?

Answer: () minutes

Q6: What is taught in the lectures regarding oral health care education in your nursing field? (multiple choice)

Answer: Preventive effect of oral health care on dental caries/Preventive effect of oral health care on periodontal disease/Preventive effect of oral health care on aspiration pneumonia/Association between periodontal disease and diabetes/Association between periodontal disease and cardiovascular disease/Usage of fluoride for dental caries prevention/Oral hypofunction/Oral frailty/Oral diadochokinesis/Perioperative oral function management

Q7: How many hours do you spend instructing on practical oral health care for your students in your nursing field?

Answer: () minutes

Q8: What is instructed in practical oral health care education? (multiple choice)

Answer: Toothbrushing technique/Usage of dental floss/Usage of an interspace brush/Usage of a dental sponge/Denture removal, cleaning, and replacement/Salivary gland massage/Oral function training (oral exercise)/Usage of personal protective equipment (PPE) during oral care practice for patients/Infection prevention measures during oral health care practice for patients other than PPE/Usage of a portable suction machine for oral health care

Q9: Who teaches oral health care in lectures in your nursing fields? (multiple choice)

Answer: Nurse/Dentist/Dental hygienist/Speech-language-hearing therapist/Others

Q10: Who teaches practical oral health care in your nursing fields? (multiple choice)

Answer: Nurse/Dentist/Dental hygienist/Speech-language-hearing therapist/Others
